# Biochemical characterization and X-ray structural and mutagenic analyses of the putative autolysin CdCwlT33800 catalytic domain from *Clostridioides difficile*

**DOI:** 10.1128/aem.01216-25

**Published:** 2025-09-16

**Authors:** Hiroshi Sekiya, Yasuhiro Nonaka, Shigehiro Kamitori, Eiji Tamai

**Affiliations:** 1 Department of Infectious Disease, College of Pharmaceutical Science, Matsuyama University 12694, Matsuyama, Ehime, Japan; 2 Department of Pharmacology, Faculty of Medicine, Kagawa University 208500, Kita-gun, Kagawa, Japan; 3 Research Facility Center for Science and Technology, Faculty of Medicine, Kagawa University 12850, Kita-gun, Kagawa, Japan; University of Milano-Bicocca, Milan, Italy

**Keywords:** *Clostridioides difficile*infection, autolysin, endopeptidase, antimicrobial agent, X-ray crystallography

## Abstract

*Clostridioides difficile* is a major pathogen of pseudomembranous colitis, and new antimicrobial agents are needed for its treatment. Autolysins are peptidoglycan-degrading enzymes that generally reorganize the cell wall during cell division but kill bacteria by bacteriolysis when applied from outside the bacterial cell. Therefore, they have potential as novel therapeutic agents for the treatment of infectious diseases. We surveyed the genome of *C. difficile* strain 630 and identified two virtually identical autolysin genes, *cdCwlT33800* and *cdCwlT*, with a lysozyme-like domain and endopeptidase domain. The entire region and each domain of the two proteins were expressed, purified, and assayed for bacteriolytic activity. Only the individual endopeptidase domain variants exhibited bacteriolytic activity against *C. difficile*. We also investigated the optimal pH and salt concentration, the effects of metal ions, thermostability, long-term storage, and the species specificity of the CdCwlT33800 endopeptidase domain (CdCwlT33800CD2). The structure of CdCwlT33800CD2 was elucidated by X-ray crystallography at a resolution of 1.45 Å. The overall structure was spherical and consisted of five helices and eight β-strands, with a 28 Å substrate-binding groove, at the center of which the catalytic residues of Cys242 and His296 were located. The structure of the substrate–enzyme complex was proposed through modeling and mutagenic analyses of CdCwlT33800CD2.

**IMPORTANCE:**

*Clostridioides difficile* is a bacterium that causes severe colitis and life-threatening diarrhea, particularly after antibiotic treatment. Since current therapies are not always effective and resistance to drugs continues to increase, there is an urgent need for new treatment strategies. One promising approach is the use of lytic enzymes, which break down the bacterial cell wall and lead to bacterial death. These enzymes include autolysins, which are produced by bacteria themselves and phage-derived endolysins. In the present study, we identified a novel autolysin from *C. difficile* and analyzed its biochemical characteristics and structure. The present results provide insights into the development of enzyme-based therapies to combat *C. difficile* infections and may lead to effective alternatives to conventional antibiotics.

## INTRODUCTION

*Clostridioides difficile* is a Gram-positive, spore-forming, anaerobic bacterium that causes antibiotic-associated diarrhea, pseudomembranous colitis, and *C. difficile*-associated diarrhea (1). Severe infections are typically treated with vancomycin or metronidazole (2), which have been shown to disrupt the gut microbiota. Fidaxomicin, a narrow-spectrum antibiotic (3), is a less disruptive alternative; however, its high cost limits its use (4). Other treatments, including fecal microbiota transplantation (5) and probiotic therapy (6), exist, but also have challenges that hinder their routine adoption (7). This underscores the need for targeted antimicrobials against *C. difficile*.

Lytic enzymes, which hydrolyze the peptidoglycans of bacterial cell walls, effectively kill bacteria. Therefore, they have potential as therapeutic agents, particularly those that exhibit species-specific lytic activity, and may serve as alternatives to standard drug therapy (8–12) and bacterial control agents in the food industry (13). Autolysins and endolysins are well-known lytic enzymes that typically have both a binding domain and catalytic domain, or only a catalytic domain, and several have more than one of these domains in a single molecule (14–17). These enzymes are categorized into four classes based on their hydrolyzing sites: glucosaminidases, muramidases, amidases, and endopeptidases. Autolysins are endogenous in bacteria and essential for various physiological processes requiring cell wall remodeling, including cell wall expansion, peptidoglycan turnover, daughter cell separation, sporulation, germination, peptidoglycan recycling, and autolysis (18–20). While phage-derived endolysins typically exhibit species specificity, autolysins generally lack this characteristic; however, some autolysins have been shown to exhibit species specificity (16). Two distinct types of species specificity have been proposed: one is based on the binding affinity of the binding domain (15), and the other is defined by the structural properties of the catalytic domain’s substrate-binding site, which governs substrate degradation specificity (16). However, the precise molecular mechanisms underlying these specificities remain unclear. A structural analysis is essential to clarify these issues. In *C. difficile*, autolysins, such as Acd24020, have been reported to induce species-specific substrate degradation, which may be attributed to the different structure of the peptide bridge in the peptidoglycans of *C. difficile* compared to those in other bacteria (16). This specificity is considered to be present in endopeptidases that recognize and cleave peptide bridges.

*C. difficile* 630 has several lytic enzyme genes in its genome (21, 22), many of which have potential as therapeutic agents for *C. difficile* infection. To date, the biochemical and/or structural characteristics of a number of these enzymes have been reported, including CdCwlT (gene ID; CD03720) (23), Ecd09610 (gene ID; CD09610) (24), Acd (gene ID; CD13040) (25), CwlA (gene ID; CD11350) (26), Ecd18980 (gene ID; CD18980) (27), and Acd24020 (gene ID; CD24020) (16), as well as several *C. difficile* phage-derived endolysins, such as CD27L, PlyCD, CDG, and CD11 (28). However, the biochemical and structural properties, reaction mechanisms, and species specificities of many other putative lytic enzymes remain largely unknown. Therefore, the clarification of their properties is important for the construction of optimal therapeutic agents. It is also biologically significant to obtain a more detailed understanding of their reaction mechanisms and species specificity.

In a survey of the *C. difficile* genome, we found two nearly identical autolysin genes (gene ID: CD33800 and CD03720). Since the protein encoded by CD03720 has been designated as CdCwlT (23), the homologous protein encoded by CD33800 was herein named CdCwlT33800. Both proteins have a lysozyme-like domain and endopeptidase domain belonging to the NlpC/P60 family. Lysozyme-like domains are a class of enzyme domains that hydrolyze the β-1,4-glycosidic bonds of peptidoglycans and are mainly found in bacterial lysozymes, endolysins (9), and some bacterial autolysins (19). Endopeptidase domains belong to the NlpC/P60 family, which cleave the γ-D-glutamyl-meso-diaminopimelic acid bond in peptidoglycans, are widely distributed among bacterial lineages, and are considered to play biologically significant roles (29). They structurally resemble the primitive papain-like cysteine peptidase, while the NlpC/P60 domain has a simpler structure consisting of one α-helix and five antiparallel β-sheets (30, 31).

We herein report the biochemical properties of a CdCwlT33800 endopeptidase domain variant, CdCwlT33800CD2, with a focus on the effects of pH, salt concentration, metal ions, temperature, and lyophilization. We also describe the structure of CdCwlT33800CD2 and its substrate-binding model.

## MATERIALS AND METHODS

### Bacterial strains and media

*Escherichia coli* (*E. coli*) DH10B, which was used as the host for plasmid construction, and *E. coli* BL21-CodonPlus-RIL, which was used for protein expression, were cultured in M9 medium containing 0.2% (wt/vol) glucose, 0.2% (wt/vol) tryptone, and 0.001% thiamine with appropriate antibiotics. *C. difficile* 630, *C. difficile* ATCC43255, *C. difficile* ATCC9689, *Clostridium acetobutylicum* (*C. acetobutylicum*) ATCC824, *Clostridium coccoides* (*C. coccoides*) ATCC29236, *Clostridium histolyticum* (*C. histolyticum*) JCM1403, *Clostridium lituseburense* (*C. lituseburense*) ATCC25759, *Clostridium novyi* (*C. novyi*) ATCC17861, *Clostridium perfringens* (*C. perfringens*) strain 13, *Clostridium ramosum* (*C. ramosum*) ATCC25582, *Clostridium tetani* (*C. tetani*) KZ1113, *Atopobium fossor* (*A. fossor*) ATCC43386, *Bifidobacterium adolescentis* (*B. adolescentis*) ATCC15703, *Eubacterium cylindroides* (*E. cylindroides*) ATCC27805, *Bacillus subtilis* (*B. subtilis*) ATCC6633, and *Staphylococcus aureus* (*S. aureus*) FDA209P were used for lytic and binding activity assays. *B. subtilis* and *S. aureus* were grown in Luria-Bertani broth at 37°C. *C. difficile* strains were grown anaerobically in Tryptone-Yeast medium containing 3% tryptone, 2% yeast extract, and 0.1% sodium thioglycolic acid at 37°C. *C. perfringens* was grown anaerobically in Tryptone-Yeast-Glucose medium containing 3% tryptone, 2% yeast extract, 0.5% glucose, and 0.1% sodium thioglycolic acid at 37°C. The other bacterial cells were grown anaerobically in Gifu Anaerobic Medium (Nissui, Tokyo, Japan).

### Construction of plasmids

**Fig 1:**
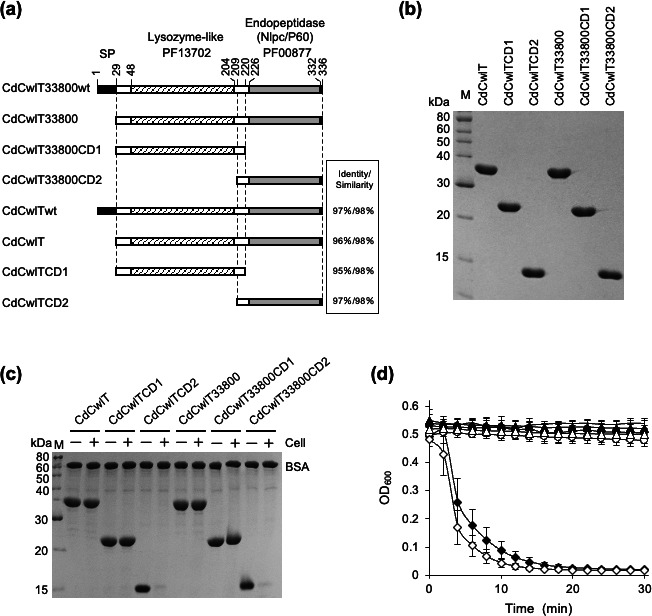
SDS-PAGE analysis, binding ability, and lytic activities of CdCwlT and CdCwlT33800 derivatives. (**a**) Schematic diagrams of CdCwlT33800wt, CdCwlT33800, CdCwlT33800CD1, CdCwlT33800CD2, CD03720wt, CD03720, CD03720CD1, and CD03720CD2 are shown. CdCwlT33800wt and CdCwlTwt are genome-encoded wild-type proteins, showing the signal peptide (SP), lysozyme-like domain (Pfam number: PF13702), and endopeptidase domain (Nlpc/P60) (Pfam number: PF00877) from the N-terminal end. CdCwlT33800CD1 and CdCwlTCD1 are mutants with only an N-terminal lysozyme-like domain, while CdCwlT33800CD2 and Acdo3720CD2 are mutants with only a C-terminal endopeptidase domain. These proteins have a His-tag at the N-terminus. The identity and similarity of the corresponding proteins of CdCwlT33800 and CdCwlT are listed on the right side. (**b**) Purified proteins (2 µg each) were analyzed by SDS-PAGE (15%). The gel was stained with Coomassie blue R. (**c**) Binding abilities of purified proteins to *C. difficile* 630 cells. The purified protein and non-binding internal standard (bovine serum albumin: BSA) were incubated without (−) or with (+) the cell. After centrifugation, the supernatant was analyzed by SDS-PAGE (15%). (**d**) The lytic activities of protein (5.0 µg) were assessed by the turbidity reduction assay against *C. difficile* 630 cells. CdCwlT (filled circles), CdCwlT33800 (open circles), CdCwlTCD1 (filled triangles), CdCwlT33800CD1 (open triangles), CdCwlTCD2 (filled diamonds), CdCwlT33800CD2 (open diamonds), and control (solid line) are shown. Lytic activity represents the average of the results of three independent experiments, each with triplicate samples.

To construct expression vectors for N-terminal His-tagged CdCwlT33800 and CdCwlT, standard PCR was performed using the primers listed in Table S1, Tks Gflex DNA Polymerase (TakaRa Bio, Inc., Shiga, Japan), and *C. difficile* 630 genomic DNA as the template. PCR products were then digested with *Nde*I and *BamH*I and cloned into the *Nde*I-*BamH*I site of the expression vector pColdII (TakaRa Bio, Inc.), yielding the plasmids pColdIICD33800 and pColdIICD03720. To construct plasmids expressing the lysozyme-like domain (pColdIICD33800CD1 and pColdIICD03720CD1) or the endopeptidase domain (pColdIICD33800CD2 and pColdIICD03720CD2), DNA fragments were amplified by standard PCR using the primers in Table S1 with pColdIICD33800 and pColdIICD03720 as templates and were then cloned into the same site of pColdII (Fig. 1a). Construction of the plasmid expressing the CdCwlT33800CD2 mutant was performed in the same manner by the overlap extension PCR method (32) using the primers shown in Table S2. PCR-amplified fragments in all constructs were verified by a sequencing analysis.

### Preparation of proteins for *in vitro* assays

*Escherichia coli* BL21-CodonPlus-RIL transformed with pColdIICD33800, pColdIICD33800CD1, pColdIICD33800CD2, pColdIICD03720, pColdIICD03720CD1, or pColdIICD03720CD2 was cultured in M9 medium containing 0.2% (wt/vol) glucose, 0.2% (wt/vol) tryptone, 0.001% thiamine, 100 µg/mL ampicillin, 30 µg/mL chloramphenicol, and 10 µg/mL tetracycline at 37°C until the middle-logarithmic phase and then incubated on ice for 30 min. After the addition of isopropyl-β-D-thiogalactopyranoside at a final concentration of 1 mM, cells were further incubated at 15°C for 20–24 h. Harvested cells were suspended in buffer A (50 mM Tris-HCl [pH 7.0], 500 mM NaCl, and 20 mM imidazole), and the suspension was sonicated on ice for five 30-second cycles at power level 5 using an ultrasonic disruptor (UD-200, TOMY Co., Ltd., Tokyo, Japan). The suspension was then centrifuged at 22,300 × *g* at 4°C for 10 min, and the supernatant was filtered using a syringe filter with a pore size of 0.2 µm. The protein solution was applied to Ni^+^-charged Chelating Sepharose Fast Flow (Cytiva, Tokyo, Japan). The column was washed with buffer A and eluted by a stepwise or linear gradient of 50–350 mM imidazole. The eluent from the resin was dialyzed against buffer B (25 mM phosphate buffer [pH 7.0], 100 mM NaCl, and 10% glycerol) and filtered through a syringe filter with a pore size of 0.2 µm.

### Lytic activity assay

The lytic activities of proteins were tested by the method of Gerova et al. with some modifications (33). Briefly, bacterial cells cultured under the above conditions were washed twice, suspended in wash buffer (25 mM Tris-HCl [pH 7.0]), and adjusted to an optical density at 600 nm (OD_600_) of 1.25 mL. Lytic activity was started by the addition of 20 µL protein or assay buffer to 180 µL of a preincubated cell suspension. OD_600_ was measured at 37°C at 1-minute intervals (SpectraMax M5e Multi-Mode Microplate Readers, Molecular Devices Corp., Sunnyvale, CA, USA).

### Cell binding assay

Cell binding assays were performed using purified CdCwlT33800, CdCwlT33800CD1, CdCwlT33800CD2, CdCwlT, CdCwlTCD1, and CdCwlTCD2 (16, 27). Heat-inactivated bacterial cells were prepared according to a previously reported method (27). Briefly, bacterial strains cultured under the conditions described above were washed with buffer containing 25 mM Tris-HCl (pH 7.0), 1 mM ethylenediaminetetraacetic acid (EDTA), and 1 mM 2-mercaptoethanol, and then ultrapure water. The cells suspended in ultrapure water were autoclaved at 121°C for 15 min. Purified proteins and bovine serum albumin were incubated on ice for 15 min either with or without heat-inactivated cells in binding buffer (25 mM Tris-HCl [pH 7.0]) (Fig. 1c). Samples were then centrifuged at 22,300 × *g* at 4°C for 3 min. SDS-PAGE sample buffer was added to the supernatant, and the mixture was incubated at 95°C for 2 min and analyzed by SDS-PAGE.

### Protein preparation and X-ray crystallography

The expression of CdCwlT33800CD2 for protein crystallization was performed using BL21-CodonPlus-RIL/pColdIICD33800CD2 under the medium and culture conditions described above. The first purification step was performed as described above using a 5 mL bed volume of HiTrap Chelating High Performance (Cytiva, Tokyo, Japan). The resulting protein was dialyzed against 50 mM 2-morpholinoethanesulfonic acid (MES) (pH 6.5), centrifuged, and filtered through a syringe filter with a pore size of 0.22 µm. The dialyzed protein was applied to a 1 mL bed volume of SP Sepharose High Performance (Cytiva, Tokyo, Japan) equilibrated with 50 mM MES (pH 6.5), washed with 50 mM MES (pH 6.5) buffer containing 200 mM dimethylethylammonium propane sulfonate (NDSB-195), and eluted with 50 mM MES (pH 6.5) containing 1 M NaCl and 200 mM NDSB-195. Desalting was performed using a PD MidiTrap G-25 column equilibrated with 50 mM MES (pH 6.5) containing 100 mM NaCl and 200 mM NDSB-195. The crystals of proteins were grown at 20°C in a droplet mixed with 1 µL of a protein solution (CdCwlT33800CD2: 23.2 mg/mL) and 1 µL of a reservoir solution (3.5 M sodium formate and 100 mM sodium acetate/HCl [pH 4.6]), against 50 µL of the reservoir solution, using the sitting drop vapor diffusion method. Data collection was performed on the PF-BL5A beam line in KEK (Tsukuba, Japan) at 100 K. Diffraction data were processed using the program XDS(34)and the CCP4 program suite (35). Data collection and scaling results are listed in Table 2. The structure of CdCwlT33800CD2 was solved by molecular replacement with the program MOLREP (36)using a model derived from the program AlphaFold2 (37). Further model building was performed with the program Coot (38), and the structure was refined using the programs Refmac5(39)and Phenix Refine(40). Refinement statistics are listed in Table 2 . Figures showing protein structures were prepared using PyMol (Schrödinger, http://www.pymol.org).

## RESULTS

### Identification, cloning, expression, and purification of putative autolysins and their derivatives

Gene IDs CD33800 and CD03720 in the *C. difficile* 630 genome were identified as putative autolysins by sequence similarity searches of endopeptidases. Since the protein encoded by CD03720 was designated CdCwlT (*C. difficile* cell wall lytic enzyme two catalytic domain) (23), the homologous protein encoded by CD33800 was named CdCwlT33800 to distinguish it from CdCwlT. These proteins had a signal peptide and lysozyme-like domain (Pfam number: PF13702) at the N-terminus and the NlpC/P60 family of the endopeptidase domain (Pfam number: PF00877) at the C-terminus (Fig. 1a). The amino acid sequences of these proteins were similar, with the identity and similarity of the entire proteins and each domain being 95%–97% and 98%, respectively (Fig. 1a; Fig. S1). The genes encoding the entire region without the signal peptide (CdCwlT33800 and CdCwlT), the lysozyme-like domain (CdCwlT33800CD1 and CdCwlTCD1), and the endopeptidase domain (CdCwlT33800CD2 and CdCwlTCD2) were cloned into an *E. coli* expression vector (pColdII) (Fig. 1a). These genes were successfully expressed with the His-Tag fused at the N-terminus and purified by Ni-affinity chromatography (Fig. 1b). To examine the binding and lytic activities of the purified proteins, binding assays (Fig. 1c) and turbidity reduction assays (Fig. 1d) were performed using *C. difficile* 630 as the substrate. In the binding assay, the protein was added to the bacterial cell suspension, and the supernatant obtained after centrifugation was analyzed by SDS-PAGE. The presence of binding activity was indicated by a decrease in the intensity of the target band from cells without this activity. In the turbidity reduction assay, if the protein exhibited bacteriolytic activity, turbidity (optical density) decreased due to bacteriolysis. Analyses of binding activities showed that the entire proteins, CdCwlT33800 and CdCwlT, and the lysozyme-like domains, CdCwlT33800CD1 and CdCwlTCD1, showed no changes in band densities in the presence or absence of bacterial cells, whereas the endopeptidase domains, CdCwlT33800CD2 and CdCwlTCD2, showed large decreases in band densities (Fig. 1c). These results indicate that a single endopeptidase domain can bind to bacterial cells. Furthermore, CdCwlT33800CD2 and CdCwlTCD2, which bind to bacterial cells, caused decreases in optical density (Fig. 1d), indicating that a single endopeptidase domain exhibited bacteriolytic activity.

### Characterization of the lytic activity of the CdCwlT33800 endopeptidase domain (CdCwlT33800CD2)

**Fig 2:**
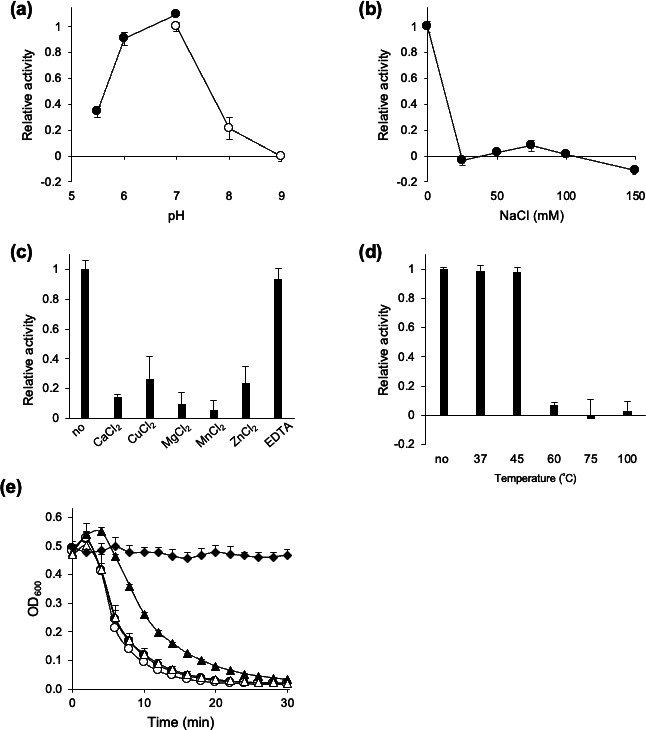
Characterization of the lytic activity of CdCwlT33800CD2 against *C. difficile* 630 using the turbidity reduction assay. (**a**) The optimal pH for the lytic activity of protein (5.0 µg) was assessed using 25 mM Bis-Tris (filled circles) and 25 mM Tris-HCl (open circles). Relative activity was expressed with lytic activity in 25 mM Tris-HCl (pH 7.0) set as 1. Lytic activity was calculated after 15 min as follows: [ΔOD_600_ test (protein added) − ΔOD_600_ control (buffer only)]/initial OD. Relative activity at 25 mM Tris-HCl (pH 7.0) was set as 1. (**b**) The effect of NaCl on lytic activity was evaluated in 25 mM Tris-HCl (pH 7.0). Relative activity at 0 mM NaCl was set as 1. (**c**) The effect of divalent metal cations on lytic activity was measured in 25 mM Tris-HCl (pH 7.0), supplemented with 1 mM CaCl_2_, MgCl_2_, ZnCl_2_, MnCl_2_, CuCl_2_, or EDTA. Relative activity at unsupplemented 25 mM Tris-HCl (pH 7.0) was set as 1. (**d**) Thermal stability of lytic activity was assessed by measuring lytic activity after 10 min of a heat treatment at 37, 45, 60, 75, or 100°C or no treatment. Relative activity with no treatment was set as 1. In these experiments, lytic activity was measured using 5 µg of protein. Means in all experiments were calculated based on three independent experiments. Standard deviations were calculated by three independent experiments, each with triplicate samples. (**e**) Effects of lyophilization on the lytic activity of CdCwlT33800CD2 (5.0 µg). An unlyophilized protein (filled circles), a lyophilized protein (open circles), a lyophilized protein stored at room temperature for four weeks (filled triangles), a lyophilized protein stored at 4°C for four weeks (open triangles), and buffer (filled diamonds) are shown. Lytic activity represents the average of the results of three independent experiments, each with triplicate samples.

Since CdCwlT33800CD2 and CdCwlTCD2, endopeptidase domains, were highly homologous, exhibited equivalent bacteriolytic activity (Fig. 1; Fig. S1), and had the same genes in their vicinity (Fig. S2), we considered them to be identical and thereafter conducted a detailed analysis of CdCwlT33800CD2. The effects of pH, salt, metal ions, temperature, lyophilization, and long-term storage on the bacteriolytic activity of CdCwlT33800CD2 were examined using *C. difficile* 630 as the substrate (Fig. 2a through e). As shown in Fig. 2a, the optimal pH of CdCwlT33800CD2 is 6–7. The addition of 25 mM NaCl abolished bacteriolytic activity, indicating that the addition of salt exerted a significant effect on this activity (Fig. 2b). The addition of CaCl_2_, CuCl_2_, MgCl_2_, MnCl_2_, and ZnCl_2_ significantly reduced lytic activity, while that of EDTA did not (Fig. 2c). In addition, to assess thermostability, the purified protein was preincubated at various temperatures for 10 min, and its activity was measured (Fig. 2d). The results obtained showed that bacteriolytic activity was retained up to 45°C, but was lost at temperatures higher than 60°C (Fig. 2d). The stability of enzymes, both long-term and under dry conditions, needs to be confirmed prior to their use as drugs. Purified CdCwlT33800CD2 was lyophilized, stored at room temperature or 4°C, and then dissolved again in water to assess its lytic activity (Fig. 2e). No reductions were observed in bacteriolytic activity with lyophilization or subsequent storage at 4°C for four weeks. However, after lyophilization and storage at room temperature for four weeks, this activity slightly decreased.

### Bacterial specificity of the CdCwlT33800 endopeptidase domain (CdCwlT33800CD2)

**Fig 3:**
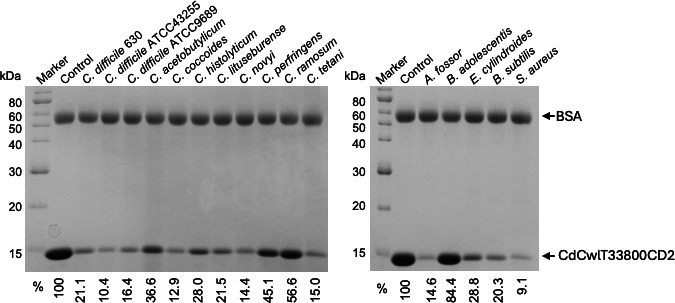
Binding assay of CdCwlT33800CD2 for various bacteria. Experiments were repeated three times with similar results. Purified CdCwlT33800CD2 and BSA were incubated on ice either with or without heat-inactivated cells, as indicated at the top of the gel. After centrifugation, supernatants were analyzed by 15% SDS-PAGE. Bands were quantified by densitometry using ImageJ, and the amount of remaining protein was expressed relative to the control (without cells), which was set at 100%, as indicated in the lower panel.

Autolysins generally do not exhibit species-specific lytic activity; however, some, particularly those in *C. difficile*, have been shown to possess it (16). Therefore, the species specificity of CdCwlT33800CD2 was assessed by a turbidity reduction assay using various bacterial strains as substrates (Table 1). These bacteria were *Clostridium* species closely related to *C. difficile*, Gram-positive gut microbiota, and representative Gram-positive bacteria. CdCwlT33800CD2 exhibited bacteriolytic activity against *C. difficile* 630 and ATCC9689 as well as *Bacillus subtilis* ATCC6633. On the other hand, it exhibited weak bacteriolytic activity against *C. difficile* ATCC43255, C. *lituseburense* ATCC25759, C. *ramosum* ATCC25582, *C. tetani* KZ1113, and *A. fossor* ATCC43386. Bacteriolytic activity was not detected against bacteria tested in this study other than those listed above. We also measured binding activity to various bacteria in order to establish whether the difference in bacteriolytic activity among bacterial species was due to binding (Fig. 3). As shown in Fig. 3, CdCwlT33800CD2 bands are reduced in most of the bacteria tested in this study; however, a smaller decrease was observed in *C. ramosum* ATCC25582 and almost no reduction in *B. adolescentis* ATCC15703. These results indicate that the species specificity of the lytic activity of CdCwlT33800CD2 was mainly dependent on the catalytic mechanism of its catalytic domain.

**TABLE 1:** Species specificity of CdCwlT33800CD2 lytic activity^*a*^

Bacteria	Relative activity (%)
	CdCwlT33800CD2
*C. difficile* 630	100.0 ± 9.80
*C. difficile* ATCC43255	31.1 ± 3.00
*C. difficile* ATCC9689	72.8 ± 16.2
*C. acetobutylicum* ATCC824	−2.7 ± 1.50
*C. coccoides* ATCC29236	−4.2 ± 5.50
*C. histolyticum* JCM1403	−3.5 ± 3.70
*C. lituseburense* ATCC25759	33.4 ± 5.80
*C. novyi* ATCC17861	9.9 ± 7.80
*C. perfringens* strain13	−0.1 ± 1.60
*C. ramosum* ATCC25582	20.0 ± 9.50
*C. tetani* KZ1113	23.4 ± 8.50
*A. fossor* ATCC43386	24.9 ± 7.80
*B. adolescentis* ATCC15703	−1.2 ± 1.10
*E. cylindroides* ATCC27805	−4.4 ± 1.00
*B. subtilis* ATCC6633	83.0 ± 1.30
*S. aureus* FDA209P	−1.7 ± 2.20

^*a*^ The bacteriolytic activity of CdCwlT33800CD2 (5 μg) against various bacteria was calculated using the strains listed in the table as a substrate and the change in turbidity (Optical density at 600 nm, OD_600_) after 15 minutes using the following formula: {ΔOD_600_ test (protein) - ΔOD_600_ control (buffer only)} / initial OD_600_. Bacteriolytic activity against various bacteria was expressed as relative activity with activity against *C. difficile* as 100%. The mean and standard deviation in all experiments were calculated by three independent experiments each with triplicate samples.

### X-ray structure of CdCwlT33800CD2

The structure of CdCwlT33800CD2 was successfully elucidated and refined to an R_cryst_ of 0.184 (R_free_ of 0.215) with a good chemical geometry using 1.45 Å resolution data (Table 2). There are two molecules, Mol-A and Mol-B, in an asymmetric unit, related by non-crystallographic twofold symmetry. The structures of the two molecules are almost identical within root-mean-square deviations of the main-chain atoms of 0.42 Å, and the structural description here concentrates on Mol-A, giving a relatively clear electron density to the whole polypeptide chain.

**TABLE 2:** Data collection and structure refinement statistics of CdCwlT33800CD2

Data collection	
Beamline/radiation source	PF BL5A
Detector	PILATUS3 S 2M
Temperature (K)	100
Wavelength (Å)	1.0000
Resolution range (Å)	30.39–1.45 (1.49–1.45)
No. of measured refs.	232,883 (14,828)
No. of unique refs.	44,782 (3,110)
Redundancy	6.3 (4.8)
Completeness (%)	96.8 (90.5)
Mean *Io*/σ*(Io*)	38.3 (8.4)
*R*_merge_ ^*a*^	0.023 (0.154)
CC_1/2_(%)	99.9 (98.2)
Space group	*R*3
Unit cell parameters	*a* = 105.26 Å, α =90°
	*b* = 105.26 Å, β =90°
	*c* = 63.10 Å, γ =120°

Refinement	
Resolution range (Å)	30.39–1.45 (1.480–1.450)
No. of refs.	44,780 (2,706)
Completeness (%)	96.8 (90.0)
*R*_cryst_ ^*b*^	0.164 (0.207)
*R*_free_ ^*c*^	0.185 (0.237)
RMSD bond lengths (Å)	0.007
RMSD bond angles (°)	1.12
Ramachandran plot	
Most favored region (%)	98.2
Allowed region (%)	1.8
*B*-factor (Å^2^)	
Protein	21.9
Solvent	31.9
PDB code	9UBF

^*a*^ *R*_merge_ = Σ*_hkl_* Σ*_i_* | *I_i_*(*hkl*) - < *I*(*hkl*)> | / Σ*_hkl_* Σ*_i_ I_i_*(*hkl*)], where *I_i_* (*hkl*) is the intensity value of the *i*th measurement of reflection *hkl* and <*I*(*hkl*)> is the mean value of *I*(*hkl*) for all *i* measurements.

^*b*^ *R*_cryst_ = Σ*_hkl_* | | *F*_obs_ | - | *F*_calc_ | | / Σ*_hkl_* | *F*_obs_ |, where *F*_obs_ and *F*_calc_ are the observed and calculated structure factors, respectively.

^*c*^ *R*_free_ is the free *R*_cryst_ for the 5% of reﬂections that were excluded from the reﬁnement.

**Fig 4:**
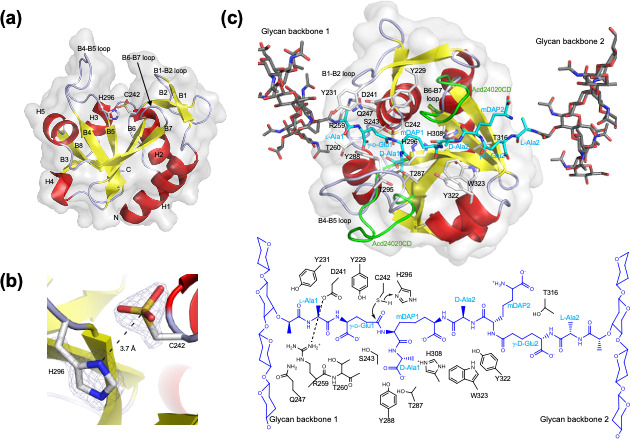
Structures of CdCwlT33800CD2. (**a**) The overall structure of CdCwlT33800CD2 is illustrated with secondary structure element labels. (**b**) The catalytic Cys242 and His296 residues are shown with the electron density of omit map at the 3.0 σ contour level. Cys242 is oxidized to cysteic acid. (**c**) The model structure of the CdCwlT33800CD2/peptidoglycan complex is shown with a schematic diagram of possible enzyme–substrate interactions. The modeled peptide side chains cross-linking two glycan backbones are illustrated. The residues of the peptide side chains and the residues of the enzyme interacting with the peptide side chains are labeled.

CdCwlT33800CD2 was spherical with five helices and eight β-strands (Fig. 4a). A large β-sheet comprising six antiparallel β-strands (B3, B4, B5, B6, B7, and B8) was noted at the center of the molecule. An α-bundle-like structure with three helices (H1, H2, and H3) and a small β-sheet formed by B1 and B2 were located on one side of a large β-sheet, while two helices (H4 and H5) were present on the opposite side. A large substrate-binding groove was detected at the center of the molecule, and a large β-sheet formed the bottom of the groove. The catalytic residues Cys242 and His296 were located at the center of the groove, facing each other, and Cys242 was oxidized to cysteic acid (Fig. 4b). The B1–B2 and B6–B7 loops formed the sidewall of the substrate-binding groove, while the B4–B5 loop formed another sidewall. H1, H2, and H3 were embedded in the molecule to stabilize the structure of the substrate-binding groove. The electron density of the B4–B5 loop of Mol-A was poor, while that of Mol-B was invisible in the final electron density map, suggesting that the B4–B5 loop was in dynamic motion.

### Modeling structure and possible interactions of CdCwlT33800CD2 with peptidoglycans

The structure of CdCwlT33800CD2 was very similar to that reported for Acd24020CD (16). Based on the X-ray structure of the Acd24020CD/citrate complex, we constructed a model structure of Acd24020-CD complexed with a peptidoglycan (16). By superimposing Acd24020-CD and CdCwlT33800CD2 in the model, the model structure of CdCwlT33800CD2 and the peptidoglycan is generated (Fig. S3a through c), as shown in Fig. 4c, with a schematic diagram of possible enzyme–substrate interactions.

CdCwlT33800CD2 had a substrate-binding groove across the molecule with a length of 28 Å (Fig. 4c), in which the B1–B2, B4–B5, and B6–B7 loops extended from both sides to cover the substrate. The peptide side chains cross-linking two glycan backbones adopted an almost extended conformation and fit the substrate-binding groove. For clarity, glycan backbones on the left and right in Fig. 4c are designated as glycan backbones 1 and 2, respectively, and the peptide side chain residues attaching to them are also given the same numbers. mDAP1 and D-Ala2 formed a cross-link of two peptide side chains, and the hydrolyzing site was located between γ-D-Glu1 and mDAP1.

The left-half moiety (L-Ala1-γ-D-Glu1-mDAP1-D-Ala1, and D-Ala2) strongly interacted with the enzyme. The catalytic Cys242(Sγ) was in contact with γ-D-Glu1(Cε) with a distance of 3.2 Å, and His296(Nδ) was directed to Cys242(Sγ) with a distance of 3.7 Å. Therefore, His296 appeared to deprotonate Cys242 to be activated as a thiolate, which nucleophilically attacked the carbonyl carbon of the scissile peptide bond to form a tetrahedral intermediate (Fig. 4c). Tyr229, Tyr231, Asp241, Ser243, Arg259, and Thr260 were in contact with the L-Ala1-γ-D-Glu1 moiety. A salt bridge between Asp241 and Arg259 contributed to the formation of a suitable substrate-binding groove. His308, Tyr322, and Trp323 were in contact with the cross-linking of mDAP1-D-Ala2 at the narrowest point of the groove. The right-half moiety (mDAP2, γ-D-Glu2, and L-Ala2) was located on the wide groove, showing a weaker interaction with the enzyme, in which only Thr316 appeared to be in contact with γ-D-Glu2. Furthermore, glycan backbone 1 appeared to be in contact with the enzyme by Tyr231 and Gln247, while glycan backbone 2 was free from the enzyme.

Marked structural differences between CdCwlT33800CD2 and Acd24020CD were found in the B4–B5 and B6–B7 loops. The B4–B5 loop of CdCwlT33800CD2 approached the substrate, and Tyr288 and Thr287 made unusually short contacts with D-Ala1. On the other hand, the B4–B5 loop of Acd24020CD (green in Fig. 4c) adopted a different conformation to widen the substrate-binding groove, avoiding a collision with the substrate. The B4–B5 loop was expected to be in dynamic motion and to change its conformation depending on substrate binding. The B6–B7 loop of CdCwlT33800CD2 was four amino acid residues shorter than that of Acd24020CD (green in Fig. 4c; Fig. S2d). The B6–B7 loop of Acd24020CD formed several interactions with mDAP1 and D-Ala2 of the substrate, while that of CdCwlT33800CD2 did not make contact with the substrate.

### Mutational analysis of CdCwlT33800CD2

To confirm the substrate–enzyme complex model proposed by X-ray crystallography and structural modeling, we performed site-directed mutagenesis targeting residues within the substrate-binding site (Table 3). Mutations at the catalytic residues Cys242 and His296 to other amino acids resulted in complete loss of enzymatic activity, confirming their essential role. Mutations at residues Tyr229, Tyr231, Asp241, Ser243, Arg259, and Thr260, which interact with the L-Ala1-γ-D-Glu1 portion of the substrate, generally led to a significant reduction in activity, with the exception of Y231F, Y231L, and T260A, which retained activity. Notably, mutations at Asp241 and Arg259, which form a critical salt bridge, markedly reduced activity, indicating the importance of this interaction for catalysis. In contrast, mutations at His308, Tyr322, and Trp323, which were located near the mDAP1–D-Ala2 region of the substrate, as well as at Thr316, located near γ-D-Glu2, did not significantly affect activity. Similarly, the mutation at Gln247, which was in contact with the glycan backbone 1, had no apparent effect. The mutation at Thr287, which interacted with D-Ala1, resulted in a marked reduction in activity, whereas the mutation at Tyr288 only caused a slight decrease. The T287A and T287Y mutants were successfully expressed and partially purified; however, they formed an insoluble inclusion body during the dialysis step (data not shown), suggesting that Thr287 also played a role in maintaining the structural stability of the protein.

**TABLE 3:** Relative activities for CdCwlT33800CD2 and mutants^*a*^

Sample	Relative activity (%）
CdCwlT33800CD2	100.0 ± 4.5
Catalytic center	
C242A	1.4 ± 4.8
C242M	−5.9 ± 7.8
C242S	−13.5 ± 8.8
C242V	0.1 ± 7.3
H296A	−10.1 ± 7.7
H296D	−3.9 ± 8.0
H296F	−2.4 ± 3.5
H296L	−15.4 ± 8.7
Substrate-binding groove	
Y229A	49.6 ± 3.9
Y229F	9.9 ± 4.2
Y229L	4.9 ± 6.1
Y229T	4.5 ± 8.9
S243A	46.3 ± 2.4
S243F	8.2 ± 4.6
S243L	11.4 ± 4.7
Y231A	41.8 ± 3.6
Y231F	100.5 ± 3.5
Y231L	71.2 ± 3.2
Y231T	15.7 ± 4.3
T260A	76.1 ± 9.8
Salt bridge	
D241A	13.1 ± 1.2
R259A	9.8 ± 6.9
R259E	4.9 ± 3.5
Contacts with γ-D-Glu20	
T316A	100.2 ± 6.1
Contacts with glycan backbone 1	
Q247A	97.8 ± 3.2
Q247L	92.8 ± 1.8
Narrowest point	
H308A	92.3 ± 2.8
Y322A	73.1 ± 10.5
W323A	98.7 ± 6.6
Contacts with D-Ala1	
T287E	3.9 ± 10.1
Y288A	75.0 ± 1.2
Y288W	56.1 ± 1.3

^*a*^ The bacteriolytic activities of CdCwlT33800CD2 and its mutant (5 μg) were calculated by the following formula using *C. difficile* 630 as the substrate and the change in turbidity (optical density at 600 nm, OD_600_) after 15 minutes: {ΔOD_600_ test (protein) - ΔOD_600_ control (buffer only)} / initial OD_600_. The activities of the mutants were expressed as relative activity with the lysis activity of CdCwlT33800CD2 as 100%. The mean and standard deviation in all experiments were calculated by three independent experiments each with triplicate samples.

## DISCUSSION

The genes *cdCwlT33800* (gene ID: CD33800) and *cdCwlT* (gene ID: CD03720), identified through a homology search for endopeptidases in the *C. difficile* 630 genome, exhibited a high degree of amino acid sequence identity (Fig. 1a; Fig. S1). Genes with similar sequences, including transposon-related elements, were found in the vicinity of both loci (Fig. S3), suggesting that these genes arose through duplication via a recombination event or the action of transposons (23). Accordingly, *cdCwlT33800* and *cdCwlT* are considered to be identical.

Both genes were predicted to contain two catalytic domains, indicative of strong bacteriolytic activity due to possible synergistic effects (41). However, the full-length proteins exhibited neither binding nor bacteriolytic activity. Furthermore, the lysozyme-like domains alone showed neither bacteriolytic nor binding activity, whereas the isolated endopeptidase domains, CdCwlT33800CD2 and CdCwlTCD2, retained both activities (Fig. 1c and d). These results suggest that intra- or inter-molecular interactions with the lysozyme-like domain inhibited the endopeptidase domain. A structural analysis of full-length CdCwlT (PDB 4HPE) showed six molecules forming three interacting pairs (Fig. S4) within the asymmetric unit (23). Quaternary structure predictions using PISA (42) indicated that these interactions formed a stable complex in which the lysozyme-like domain blocked the catalytic site of the endopeptidase. However, mixing the two domains as separate polypeptides did not suppress activity (data not shown), implying that the inhibitory effect was mediated by intra- or intermolecular interactions between the two domains in the full-length protein. Therefore, the lysozyme-like domain may serve as a regulatory element of binding and catalytic activities.

CdCwlT33800CD2 exhibited specificity against *C. difficile*, and its activity was similar to previously reported *C. difficile*-specific bacteriolysis enzymes (16, 24, 35). However, its activity was significantly reduced in the presence of salt and metal ions, indicating limitations for direct therapeutic use. Nevertheless, the enzyme retained activity following lyophilization and during long-term storage at low temperatures, which are properties that are favorable for formulation. Therefore, while genetic modifications will be necessary to overcome its limitations, CdCwlT33800CD2 may be a promising agent against *C. difficile* infections.

Structural information is essential for improving protein function via mutagenesis. The structure of CdCwlT33800CD2 elucidated at 1.45 Å resolution closely resembled the corresponding domain in full-length CdCwlT (PDB 4HPE) (23) and the catalytic domain of Acd24020 (PDB 7CFL) (16), another *C. difficile*-specific autolysin. A substrate-binding model (Fig. 4; Fig. S2) was constructed based on Acd24020CD and confirmed through site-directed mutagenesis. Most mutations targeting residues presumed to form the substrate-binding groove exhibited significant loss of activity. However, some mutants, namely, Y231F, Y231L, Q247A, Q247L, T260A, Y288A, H308A, T316A, Y322A, and W323A, retained activity (Table 3). The Cys242 and His296 mutants completely lost activity, and an alignment analysis confirmed these residues as catalytic centers (Fig. S1). Although Cys242 was oxidized to cysteic acid in the crystal structure, based on the proposed degradation mechanism involving a nucleophilic attack by cysteine (Fig. 4c), Cys242 was presumed to function in its reduced form, as has also been suggested for YkfC (31). The retained activity observed in some mutants may reflect structural or functional redundancy. For example, the activity of Y231F suggests that a hydrophobic aromatic residue at this position is sufficient. Thr260 may lie outside the critical binding groove, and alanine substitutions at His308, Yyr322, and Trp323 may not impair activity due to reduced steric hindrance. Gln247 may interact with the glycan backbone, but did not appear to be essential for substrate recognition. Collectively, structural and mutagenesis data support the validity of the proposed substrate-binding model, particularly with respect to the location of the catalytic center and the cleavage site in the peptidoglycan substrate. Furthermore, it has been suggested that the peptide bridge must conform to the shape of the substrate-binding groove and the cleavage site must be correctly aligned with the cysteine–histidine catalytic dyad in order for the catalytic domain of CdCwlT33800 to exhibit enzymatic activity. Bacterial strains that were bound, but not lysed, by CdCwlT33800CD2 (e.g., *C. coccoides*, *C. histolyticum*, *S. aureus*, and *E. cylindroides*, as shown in Fig. 3), which possess different peptide bridges from *C. difficile*, are presumed to possess peptide bridges that are compatible with binding by CdCwlT33800CD2; however, lysis appeared to fail due to the misalignment of the cleavage site relative to the catalytic residues. Collectively, these results suggest that, similar to the species-specific mechanism observed in Acd24020 (16), precise complementarity between the shape of the substrate-binding groove and that of the substrate is considered an important factor affecting species specificity.

## Data Availability

Data that support the results of this study are available from the corresponding authors upon reasonable request.

## SUPPLEMENTAL MATERIAL

Supplemental material aem.01216-25-s0001.pdf Tables S1 and S2; Fig. S1 to S4.

ASM does not own the copyrights to Supplemental Material that may be linked to, or accessed through, an article. The authors have granted ASM a non-exclusive, world-wide license to publish the Supplemental Material files. Please contact the corresponding author directly for reuse.
