# Supplementary material for: Biochemical characterization and X-ray structural and mutagenic analyses of the putative autolysin CdCwlT33800 catalytic domain from Clostridioides difficile
Source: Appl Environ Microbiol. 2025 Sep 16;91(10):e01216-25. doi: 10.1128/aem.01216-25 (PMC12542765; doi:10.1128/aem.01216-25)
Supplement: Supplemental material — Tables S1 and S2; Fig. S1 to S4. [file aem.01216-25-s0001.pdf]

Table S1 Primers used to construct CdCwlT33800 and CdCwlT and their catalytic domain mutant expression vectors.

Primer name	Primer sequence (5'- 3')
CD33800_N	CGCcatatgATGGCGGACAGCGACG
CD33800_C	CGCggatccCTATTGTTTTACTCGTCCTG
CD03720_N	CGGcatatgGCGGACAGCGACGACGAGAACAGCAACTTTTCTTCTGGC
CD03720_C	CGCgggatccCTATTGTTTAACTCGTCCTGC
pColdF2	GTAAGGCAAGTCCCTTCAAGAG
pColdR	GGCAGGGATCTTAGATTCTG
CD33800_3R-1	CGCggatccTTATTCATTCATTACCTTTTGTGC
CD33800_CD2	CGGcatatgGTATTGGGAGAGCTGGC
CD03720_3F_2	CGGcatatgGTATCGGGAGAACTGGC

Lower-case letters indicate the restriction enzyme site. To code the entire regions of CdCwlT33800 and CdCwlT, CD33800_N and CD33800_C, and CD03720_N and CD03720_C were used, respectively. To clone CdCwlT33800CD1 and CdCwlT33800CD2, pColdF2 and CD33800_3R-1, and CD33500_CD2 and pColdR were used, respectively. To clone CdCwlTCD1 and CdCwlTCD2, pColdF2 and CD33800_3R-1, and CD33500_3F_2 and pColdR were used, respectively.

Table S2 Primers used to construct the CdCwlT33800CD2 mutant.

Primer name	Primer sequence (5'- 3')
pColdF2	GTAAGGCAAGTCCCTTCAAGAG
pColdR	TGGCAGGGATCTTAGATTCTG
CD33800-Y229AT-F	AGGCTGGAAGrcTGTATATGGCG
CD33800-Y229FL-R	CGCCATATACwaACTTCCAGCCT
CD33800-Y231AT-F	GAAGTATGTArcTGGCGGTAGTA
CD33800-Y231FL-R	TACTACCGCCAarTACATACTTC
CD33800-F240AD-F	GAACACTTCCgmTGACTGTTCGG
CD33800-D241AN-R	GTCCCGAACAGkCAAAGGAAGTG
CD33800-C242STA-F	TTCCTTTGACdcTTCGGGACTTA
CD33800-C242MV-R	TAAGTCCCGAcAyGTCAAAGGAA
CD33800-S243A-F	CTTTGACTGTgCGGGACTTACGC
CD33800-S243FL-R	GCGTAAGTCCwaaACAGTCAAAG
CD33800-Q247A-F	GGACTTACGgcATGGTGCTAT
CD33800-Q247L-R	ATAGCACCATaGCGTAAGTCC
CD33800-R259AE-F	TCTTTACCTgmAACAGCACAAATG
CD33800-T260A-R	CATTTGTGCTGcTCTAGGTAAAGA
CD33800-T287AE-F	TTCCATTCCgmaTATAACGCTGGT
CD33800-T287Y-R:	ACCAGCGTTATAGtaGGAATGGAA
CD33800-Y288A-F	TTCCATTCCACCgcTAACGCTGGT
CD33800-Y288W-R	ACCAGCGTTccaGGTGGAATGGAA
CD33800-H296AD-F	GTATGTAACCgmCGTCGGTATCT
CD33800-H296FL-R	AGATACCGACwaaGGTTACATAC
CD33800-H308A-R	GGTCGCCTGCAgcGTACATCTGA
CD33800-T316A-F	ATAGGATATgCAGACCTAAGT
CD33800-T316A-R	ACTTAGGTCTGcATATCCTAT
CD33800-Y322A-F	AGTAGTAGTgcCTGGCAACAGCAC
CD33800-W323A-R	GTGCTGTTGCgcGTAACTACTACT

Lower-case letters indicate mutated nucleotides.

Mixed bases are indicated using the following notation:

r: A or G, w: A or T, m: A or C, s: C or G,

The gene for each mutant was constructed using pColdF2 and the reverse primer (-R), and pColdR and the forward primer (-F) in the first PCR, followed by pColdF2 and pColdR in the second PCR. After ligation, transformation was performed, and the plasmid was prepared from the resulting colony and transformed again. The candidate plasmids obtained were sequenced by a sequencing analysis.

Supplemental Figure S1

(a)

```
              Accession no.
                                                                      *
PF13702                          1 SEEVLAYQPMVEKEAKEQGIPEYVPLILAIIYQESKG-KGGDVMQSSESLGGPPNTID-P
CdCwlT33800   CAJ70282.1        48 SAEVLKHQPMVEKYARENGISEYVNVLLAIIQVESGG-TAEDVMQSSESLGLPPNSLD-M
CdCwlT        CAJ67194.1        48 SAEVLKHQPMVEKYARENGISEYVNVLLAIIQVESGG-TAEDVMQSSESLGLPPNSLD-T
Pmp23         WP_000476367.1    28 VKQVMTYQPMVREILSEKDTPANEELVLAMIYTETKG-KEGDVMQSSESASGSTNTINDN
NLP/P60       pdb|4FDY|A        23 SPEVLAHRPLIEKYGKEYGIEDYVSYILAIXQVESGG-TAEDVXQSSESLGLPPNSLS-T
CwlT          NP_388378.1       54 APEVERFRAVFEKYARQEGVFDQVNIIMALTMQESGG-RSLDIMQSSESIGLPPNSITDP
Lysozyme      WP_017895794.1    38 PAAVQKWRPLVSEVANKYKVGNYTEVLLAIVYQESGDTGTNDIMQSSESEGLPPNSIVDP

              Accession no.
PF13702                         59 EESIKQGVKYLAENLEKAKKKGVD-LWTAAQAYNFGKGYIDYVAENGGKHTEELAKQYSK
CdCwlT33800   CAJ70282.1       106 ESSIKQGCKYFASLLSSCKNQGIDDLNVAIQSYNIGGGYVGYVAGKGKKHTYNLAESFAR
CdCwlT        CAJ67194.1       106 ESSIKQGCKYFASLLSSSKNQGIDDLNVAIQSYNIGGGYVGYVAGKGKKHTFNLAESFAR
Pmp23         WP_000476367.1    87 ASSIRQGVQTITDNLYLAQKKGVD-VWTAAQAYNFGPAYIDFIAQNGKENTLALAKQYSR
NLP/P60       pdb|4FDY|A        81 EESIKQGVKYFSELLTSAEQQGVD-IDSVIQSYNIGGGFLNYVRSHGKKYTYELAEQFSK
CwlT          NP_388378.1      103 ERSIEVGIKHFKKVFKQA---GGD-VRLTLQAYNFGSGFIDYVKKNGGKYTKKLALDFSR
Lysozyme      WP_017895794.1    98 ETSINVGIKYFSSIVQYADKKKCD-LATIIQSYNFGQGYVDYVASNGGKSTKELAESFSA

              Accession no.
PF13702                        118 EVV-------TGEKYTYSNPVAIEYNGGWLYAYGNIFYAEHVKQY
CdCwlT33800   CAJ70282.1       166 EKS-------GGKKVTYTNPIAVVKNGGWRYQYGNQFYVELVNQY
CdCwlT        CAJ67194.1       166 EKS-------GGKKVTYTNPIAVAKNGGWRWNYGNMFYVELVNQY
Pmp23         WP_000476367.1   146 DTVAPLLGNTTGKTYSYIHPISIFHGAELYVNGGNYYYSRQVQLN
NLP/P60       pdb|4FDY|A       140 EKS-------GGQKADYPNPIAIPVNGGWRYNYGNQFYVQLVSQY
CwlT          NP_388378.1      169 LQAFKMGWK----------------------SYGDPSYVDHVMRY
Lysozyme      WP_017895794.1   147 SH-----GG----------------------GYGDPSYYDHVMRY
```

(b)

```
              Accession no.
                                                     *
PF00877                          1 GVPYRWGGGSP-SGFDCSGLVRYAFA-KVGIELPRSSGQQY--NAGKKTIPKSEPQRGDL
CdCwlT33800   CAJ70282.1       226 GWKYVYGGSNPNTSFDCSGLTQWCYG-KAGISLPRTAQMQY---DATQHLPLSQAKAGDL
CdCwlT        CAJ67194.1       226 GWKYVYGGSNPNTSFDCSGLTQWCYG-KAGISLPRTAQAQY---DATQHLPLSQAKAGDL
Ecd09610      CAJ67802.1       542 GKPYAWGGNGP-KSFDCSGLMVWAFKRGAGINLKRVSADQSKDSRGKLLCNINDVKAGDL
Acd24020      CAJ69287.1       284 GKPYVWGAEGP-NSFDCSGFTQYVMKKSVGVSIPRVSRDQ---SKYGTYVNRGDLRSGDL
LytE          WP_305090582.1   232 GTPYKWGGTT-TSGFDCSGFIWYVLNKQTSVGRTSTAGYWSSMKS------IASPSVGDF
NlpC          CAD6006132.1      47 GTPYRYGGMTR-RGVDCSGFVVVTMRDRFDLQLPRETKEQA--SIGT-QIDKDELLPGDL
PA3472        AAG06860.1        75 GTPYRWGGTTPKKGFDCSGLVNYVFQDVDDVDLPRTARAIYNMDN--NKVSRGKLQPGDL
YafL          CAD6016180.1     134 GKPYVWGGTRPDQGFDCSGLVFYAYNKILEAKLPRTANEMYHYHRA-TIVANNDLRRGDL
YdhO          CAD6006475.1     153 GKPYRWGGSSPRTGFDCSGLVYYAYKDLVKIRIPRTANEMYHLRDA-APIERSELKNGDL
YkfC          CAA05579.1       185 GLPYLWGGISG-FGFDCSGFMYSIFKAN-GYSIPRDAGDQ---AKAGKVVPLDDMKAGDL

              Accession no.
                                                   *
PF00877                         57 VFFGTGKGI----SHVGIYLGNGQMLHASTGGG--VSISSINGGYWQKR-LVGVRR---
CdCwlT33800   CAJ70282.1       282 VFFHSTYNAGSYVTHVGIYVGNNQMYHAGDP----IGYTDLSSSYWQQH-LIGAGRVKQ
CdCwlT        CAJ67194.1       282 VFFHSTYNAGSYVTHVGIYVGNNQMYHAGDP----IGYADLSSSYWQQH-LIGAGRVKQ
Ecd09610      CAJ67802.1       601 VFFAYN-KGKGNVHHVGLYIGNDQYIHAPQTGD-VVKISSLSGRQKKKHDFARARRFF-
Acd24020      CAJ69287.1       340 VFFDTQGSNNGSVSHVGIYIGNGDMIHASSGSSKKVTISNINSSYY-SSRYVNARRVL-
LytE          WP_305090582.1   285 VFFTTYKSG---PSHMGIYIGNNNFIHAGSDG---VQISSINNSYWKPR-YLGAKRF--
NlpC          CAD6006132.1     103 VFFKTGSGQ--NGLHVGIYDTNNQFIHASTSKG--VMRSSIDNVYWQKN-FWQARRI--
PA3472        AAG06860.1       133 VFFRIRSRS---VDHVGIYVGNDRFVHAPRRGK-KVRVSDINSSYWKRH-YLAGKRILP
YafL          CAD6016180.1     193 LFFHIHSRE--IADHMGVYLGDGQFIESPRTGE-TIRISRLAEPFWQDH-FLGARRILT
YdhO          CAD6006475.1     212 VFFRTQGRG--TADHVGVYVGNGKFIQSPRTGQ-EIQITSISEDYWQRH-YVGARRVMT
YkfC          CAA05579.1       240 LFFAYE-EGKAIHHVGLYVGGGKMLHSPKTGK-SIEILTITETIYEKE-LCAVRRCFS
```

Figure S1. Alignment of CdCwlT33800 domains. The amino acid sequences of the (a) lysozyme-like (PF13720) and (b) endopeptidase (PF00877: NlpC/P60 family) domains are shown. The alignment of sequences was conducted using Clustal Omega (https://www.ebi.ac.uk/Tools/msa/clustalo/). Accession no.: NCBI GenBank accession number. Conserved and identical residues (more than half) are shaded in gray and black, respectively. Asterisks indicate catalytic residues.

Supplemental Figure S2

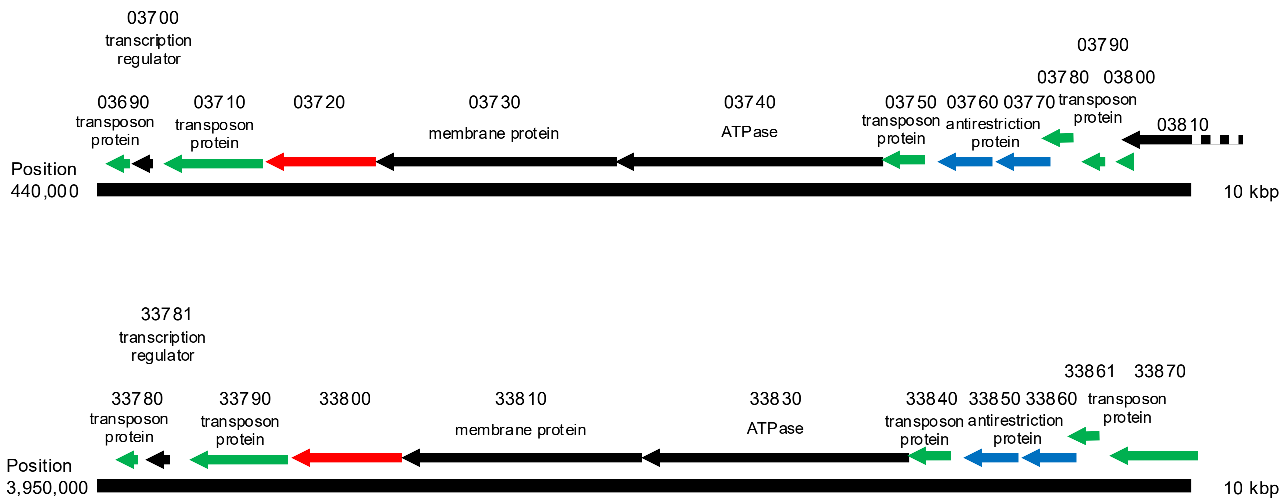

Figure S2. Genes in the vicinity of CD33800 and CD03720 genes. Genes (arrows) in the vicinity of the CD03720 gene (upper panel) and CD33800 gene (lower panel) in the C. difficile 630 genome are shown, with the gene number and protein classification.

Supplemental Figure S3

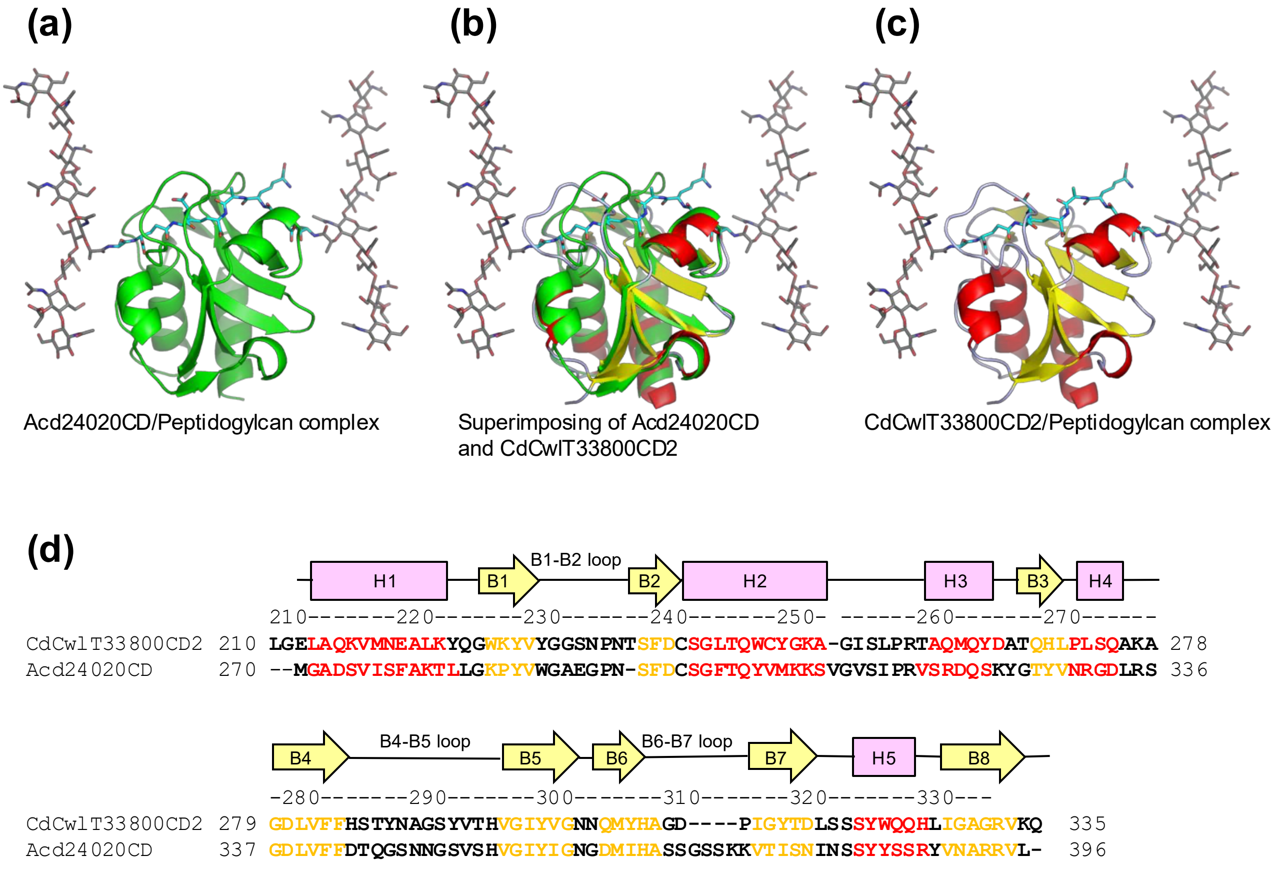

Figure S3. The procedure to generate the model structure of the CdCwlT33800CD2/peptidoglycan complex. (a) The model structure of the Acd24020CD/peptidoglycan complex is shown (16). (b) The structure of CdCwlT33800CD2 was superimposed on the structure of Acd24020CD using the secondary structure. (c) The model structure of the CdCwlT33800CD2/peptidoglycan complex was generated. (d) Amino acid sequence alignment of CdCwlT33800CD2 and Acd24020CD based on the matching of three-dimensional structures is shown with a secondary structure element label.

Supplemental Figure S4

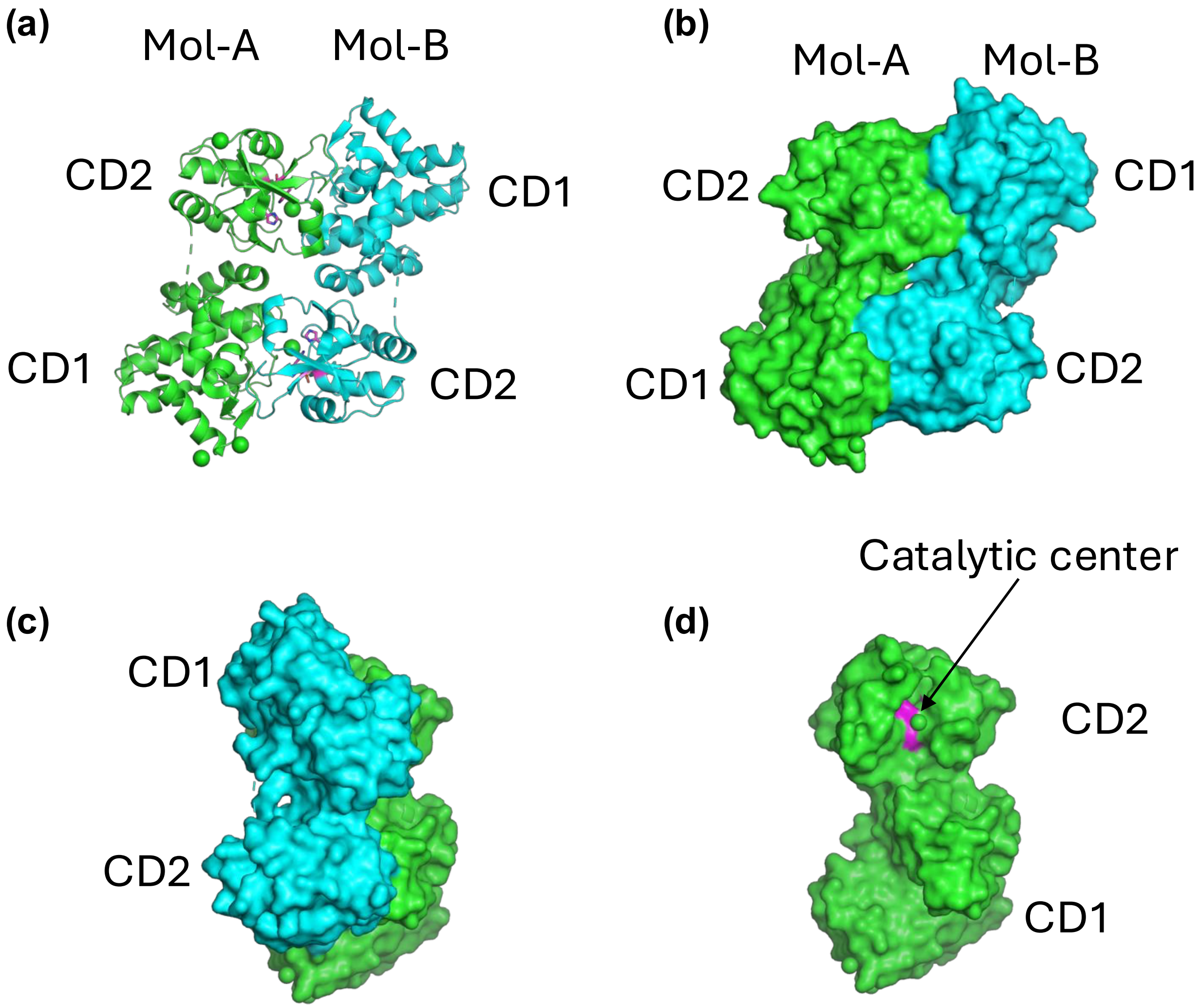

Figure S4. The entire structure of CdCwlT as registered in the Protein Data Bank. Mol-A (green) and Mol-B (blue) out of the six molecules of CdCwlT in the asymmetric unit are shown in the ribbon model (a) and surface model (b). The lysozyme-like domain (CD1) and endopeptidase domain (CD2) of each molecule are also shown. The figure was rotated (b) 90° horizontally so that Mol-B is in the front (c). MolB was removed from Figure (c) and the catalytic center (pink) is indicated by an arrow (d). These data are registered by the Joint Center for Structural Genomics (JCSG) in the Protein Data Bank and the registration number is 4HPE.
